# Neuroplastic changes in resting-state functional connectivity after stroke rehabilitation

**DOI:** 10.3389/fnhum.2015.00546

**Published:** 2015-10-05

**Authors:** Yang-teng Fan, Ching-yi Wu, Ho-ling Liu, Keh-chung Lin, Yau-yau Wai, Yao-liang Chen

**Affiliations:** ^1^School of Occupational Therapy, College of Medicine, National Taiwan University and Division of Occupational Therapy, Department of Physical Medicine and Rehabilitation, National Taiwan University HospitalTaipei, Taiwan; ^2^Department of Occupational Therapy and Graduate Institute of Behavioral Sciences, College of Medicine, Chang Gung UniversityTaoyuan, Taiwan; ^3^Healthy Aging Research Center, Chang Gung UniversityTaoyuan, Taiwan; ^4^Department of Imaging Physics, Division of Diagnostic Imaging, The University of Texas MD Anderson Cancer CenterHouston, TX, USA; ^5^Department of Medical Imaging and Radiological Sciences, Chang Gung UniversityTaoyuan, Taiwan; ^6^Department of Physical Medicine and Rehabilitation, Division of Occupational Therapy, National Taiwan University HospitalTaipei, Taiwan; ^7^Department of Diagnostic Radiology, Chang Gung Memorial HospitalKeelung, Taiwan; ^8^MRI Center, Chang Gung Memorial HospitalTaoyuan, Taiwan

**Keywords:** functional connectivity, stroke, rehabilitation, robot, fMRI, motor cortex

## Abstract

Most neuroimaging research in stroke rehabilitation mainly focuses on the neural mechanisms underlying the natural history of post-stroke recovery. However, connectivity mapping from resting-state fMRI is well suited for different neurological conditions and provides a promising method to explore plastic changes for treatment-induced recovery from stroke. We examined the changes in resting-state functional connectivity (RS-FC) of the ipsilesional primary motor cortex (M1) in 10 post-acute stroke patients before and immediately after 4 weeks of robot-assisted bilateral arm therapy (RBAT). Motor performance, functional use of the affected arm, and daily function improved in all participants. Reduced interhemispheric RS-FC between the ipsilesional and contralesional M1 (M1-M1) and the contralesional-lateralized connections were noted before treatment. In contrast, greater M1-M1 functional connectivity and disturbed resting-state networks were observed after RBAT relative to pre-treatment. Increased changes in M1-M1 RS-FC after RBAT were coupled with better motor and functional improvements. Mediation analysis showed the pre-to-post difference in M1-M1 RS-FC was a significant mediator for the relationship between motor and functional recovery. These results show neuroplastic changes and functional recoveries induced by RBAT in post-acute stroke survivors and suggest that interhemispheric functional connectivity in the motor cortex may be a neurobiological marker for recovery after stroke rehabilitation.

## Introduction

Resting-state functional magnetic resonance imaging (RS-fMRI) has emerged as a powerful tool for evaluating intrinsic brain connectivity and regional interactions during wakeful rest (Friston, 1994; Raichle and Mintun, 2006; Van Essen et al., 2012). The advantage of RS-fMRI is that it can be performed without task demand or external input and is therefore particularly useful for investigating connectivity mapping in patients with different levels of neurological impairment (Park et al., 2011).

Resting state functional connectivity (RS-FC) reflects the strength of temporal synchrony of blood oxygen level—dependent signals between spatially remote brain regions (Biswal et al., 1995; Gusnard and Raichle, 2001) and has been applied in stroke survivors (Rehme and Grefkes, 2013). Widespread changes in network functional connectivity take place immediately after stroke and have important implications for recovery (Carter et al., 2012; Grefkes and Ward, 2014). RS-FC research shows that the functional connectivity between the ipsilesional and the contralesional primary sensorimotor cortex is significantly diminished at the early stage of stroke (Wang et al., 2010; Park et al., 2011; Golestani et al., 2013). Decreased functional connectivity with the ipsilesional primary motor cortex (M1) was also found in other brain regions such as the bilateral supplementary motor area (SMA), bilateral secondary somatosensory cortex, bilateral cerebellum, bilateral thalamus, contralesional premotor cortex, and contralesional posterior parietal cortex (Carter et al., 2010; Wang et al., 2010).

During the recovery process after stroke, the resting state interhemispheric connectivity between the bilateral M1 increases (Wang et al., 2010; Park et al., 2011; Golestani et al., 2013), which is associated with motor improvements (Carter et al., 2010; Varkuti et al., 2013; Xu et al., 2014) and can predict better upper limb (UL) motor improvements in the next 6 months after stroke (Park et al., 2011). Seminal research on treatment-induced RS-fMRI found that increases in functional connectivity between the sensorimotor areas were correlated with gains in the Fugl-Meyer Assessment (FMA) score and improvements in the performance of activities of daily living (ADL) (James et al., 2009; Varkuti et al., 2013; Young et al., 2014). These imply that RS-FC changes may modulate motor and functional recovery after stroke and provide preliminary neural evidence for movement and functional improvements after a rehabilitation intervention. Notably, the brain is primed to neurological recovery in the first 3 months after stroke (Kwakkel et al., 2004; Murphy and Corbett, 2009), and most of the existing treatment-induced RS-FC studies have paid scant attention to the post-acute stage of stroke.

Robot-assisted bilateral arm therapy (RBAT), one of the main approaches to bilateral arm training, has gained increasing popularity in stroke neurorehabilitation and shows promising results in improving recovery of post-stroke sensorimotor functions (Hesse et al., 2005; Lin et al., 2010; Basteris et al., 2014; De Santis et al., 2014; Hughes et al., 2015). Enhanced motor function of stroke patients after receiving RBAT was reflected by results on the FMA and the Wolf Motor Function Test (WMFT) at the end of treatment and/or at the 3-month follow-up (Liao et al., 2012; Wu et al., 2013; Hsieh et al., 2014). A few studies have examined the changes in interhemispheric ipsilesional and contralesional M1 (M1-M1) functional connectivity but focused on the administration of *unilateral* robot-assisted therapy. Results showed that interhemispheric M1-M1 connectivity was increased in patients receiving unilateral robot-assisted therapy compared with pre-treatment and that a greater improvement in the pre-to-post difference in M1-M1 RS-FC was associated with greater gains in motor function (Sergi et al., 2011; Saleh et al., 2012).

These findings on unilateral robot-assisted therapy imply that interhemispheric motor cortex connectivity during the resting state may be a potential marker of stroke recovery after rehabilitative therapies (Chen and Schlaug, 2013). Nevertheless, the connectivity patterns after unilateral robot-assisted therapy may be different from RBAT because these two approaches induce different effects on stroke recovery (Van Delden et al., 2012; Wu et al., 2013). To our best knowledge, no study has looked at the effect of *bilateral* robot-assisted training on the functional connectivity features of the involved neuronal network during the resting state. There are only task-based neuroimaging studies showing that bilateral training facilitates excitability of transcallosal projections from the ipsilesional to the contralesional M1 (Stinear et al., 2014) and induces greater activation in the ipsilesional motor-related areas (Waller et al., 2014) than unilateral training. Illuminating the functional connectivity patterns in more regions and at the resting state post-stroke RBAT is of crucial importance, because these patterns might be potential markers of stroke rehabilitation and influence outcomes after stroke.

Previous research reveals that the recovery of the UL motor impairments is associated with the performance of ADL in stroke survivors (Dromerick et al., 2006; Wei et al., 2011) and that motor scales, such as the FMA and the WMFT, have good predictive validity with the Functional Independence Measure (FIM) at post-stroke rehabilitation (Hsieh et al., 2009). In addition, brain-imaging research has found that interhemispheric functional coherence in the resting state, particularly between the primary motor cortices, affects cortical reorganization and functional recovery after stroke (Rehme and Grefkes, 2013; Grefkes and Ward, 2014). Although these findings suggest that the change of M1-M1 RS-FC is linked with motor and functional outcomes during stroke recovery, the possibility that these neurological and behavioral variables may be interrelated, especially post-stroke RBAT, has not been tested. Thus, if our data show associations between the changes in clinical outcomes and pre-to-post difference in RS-FC after intervention, we would use a mediation analysis, a* post hoc* strategy, to further explore whether intrinsic brain connections represent a mediator between motor and functional recovery.

In the present study, we used seed-voxel correlation mapping (Horwitz et al., 1998) to investigate the changes of interhemispheric RS-FC in stroke survivors receiving RBAT. In addition to clinical measures for motor and functional outcomes, the relationships between RS-FC changes and motor and daily functions were also examined. We hypothesized that such interhemispheric connectivity would increase, that motor and functional performance would improve from pre-treatment to the end of treatment, and that the increase in RS-FC would correlate with improvements in motor performance and functional independence. Further, we would conduct a mediation analysis to test whether the pre-to-post difference in interhemispheric RS-FC is a significant mediator for an association between motor and ADL outcomes.

## Materials and Methods

This study was approved by the local Ethics Committee and conducted in accordance with the Declaration of Helsinki.

### Participants

The study enrolled 10 participants (8 men; mean age, 52.7 ± 6.5 years) with post-acute stroke in the right (*n* = 7) or left (*n* = 3) hemisphere. All participants gave written informed consent for the study. The diagnosis of stroke was clinically confirmed by computed tomography scanning. The average time from stroke onset was 46.8 (standard deviation, 20.11) days.

The inclusion criteria were: (1) first episode of stroke in cortical or subcortical regions; (2) time since stroke of less than 3 months and more than 2 weeks; (3) no serious cognitive impairment (Mini Mental State Exam score > 24) (Teng and Chui, 1987); and (4) initial motor part of the FMA-upper limb (FMA-UL) score ranging from 24–52 (Fugl-Meyer et al., 1975). We chose participants with mild and moderate limb paresis because they may have beneficial results with robot-assisted training (Housman et al., 2009; Hsieh et al., 2012). Exclusion criteria were: (1) aphasia that might interfere with understanding instructions; (2) chronic inflammatory, autoimmune, and hematologic disorders; (3) intake of anti-inflammatory drugs; (4) other major health problems or poor physical condition that might limit participation; and (5) current participation in any other research. Participants’ background information is reported in Table 1.

**Table 1:** **Demographic and clinical characteristics of right-handed study participants**.

							FMA-UL		WMFT-FAS		FIM	
Patient	Age (y)	Sex	Time from stroke (d)	Type of stroke	Lesion side/site	Laterality index (%)	Pre	Post	Pre	Post	Pre	Post	Rehabilitation procedures
1	52	Male	23	Ischemic	R/BG, CR, Th, BS	100	25	38	2.40	2.80	80	88	PT, acupuncture
2	49	Male	22	Ischemic	R/Th	100	50	61	3.00	3.53	103	115	PT, acupuncture
3	60	Male	35	Ischemic	L/F, Pu	100	33	64	2.20	3.53	101	119	PT, acupuncture
4	44	Male	63	Ischemic	R/Th, BS	100	32	52	2.00	2.53	81	90	PT
5	42	Female	58	Ischemic	R/Pu, Th	100	52	63	3.75	4.00	80	82	PT, acupuncture
6	61	Male	17	Ischemic	R/IC	80	20	31	1.67	2.00	91	96	PT, acupuncture
7	52	Male	60	Hemorrhagic	L/Pu, IC	100	27	40	2.20	2.53	81	87	PT, acupuncture
8	58	Male	61	Hemorrhagic	R/F, Pu	100	38	48	2.73	3.10	93	100	PT, acupuncture
9	51	Female	60	Ischemic	R/IC, Th	100	45	56	2.93	3.27	89	92	PT, acupuncture
10	58	Male	69	Hemorrhagic	L/Pu	100	48	60	3.53	3.80	106	109	PT, acupuncture

Abbreviations: R, right hemisphere lesion; L, left hemisphere lesion; BG, basal ganglia; CR, corona radiate; Th, thalamus; BS, brain stem; IC, internal capsule; P, putamen; F, frontal; FMA-UL, the Fugl-Meyer Assessment-upper limb; WMFT-FAS, the Wolf Motor Function Test-functional ability scores; FIM, the Functional Independence Measure; PT, physical therapy.

### Procedure

Eligible participants received RBAT. Motor performance, functional use of the affected arm, ADL, and the RS-fMRI were assessed at pre-treatment and at the end of 4 weeks of RBAT by the same blinded rater. All assessments were done within 3 days of each timestamp. The blinded raters were trained to properly administer these measures, and 2 certified occupational therapists assessed the raters’ competence.

### Intervention Protocols

The RBAT was implemented for 90 min/day, 5 days/week, for 4 weeks. All participants received 5 min of tone normalization for the arm at the beginning of therapy. Training was administrated during regularly scheduled occupational therapy, and all other routine interdisciplinary rehabilitation that did not focus on UL training was continued as usual.

Participants received 70 min of RBAT. They practiced with the Bi-Manu-Track (Reha-Stim Co, Berlin, Germany), which includes wrist flexion-extension and forearm pronation-supination movements. During the Bi-Manu-Track training, participants used their nonparetic and paretic hands under active and passive modes.

- In mode 1, both arms were guided passively by the device.
- In mode 2, the paretic arm guided the nonparetic arm and/or was guided by the nonparetic arm, depending on the participant’s level of motor severity.
- In mode 3, the nonparetic arm had to overcome continual resistance through the entire movement, and the paretic arm had to overcome only the initial resistance, which was set by the therapist according to the resistance against which the participant performed the voluntary movement with maximal force.

The robot was equipped with a computer game to provide instant visual movement feedback and to increase motivation.

The RBAT was followed by 15 min of functional task practice, which included various unilateral tasks and/or bilateral tasks such as picking up coins and opening a jar with one hand stabilizing while the other hand manipulated.

### Clinical Measures

We used changes in motor impairment and motor function on the FMA-UL and the WMFT to evaluate motor recovery. The FMA is a widely used quantitative measure of sensorimotor conditions in stroke survivors. Each item was rated on a 3-point ordinal scale, with 2 points indicating performed completely, 1 point was given for partial performance, and 0 indicated cannot perform. The 33 UL items were used to measure the movement and reflexes of the shoulder, elbow, forearm, wrist, and hand, and coordination (Appendix 1). The FMA motor subscale shows high reliability, validity, and responsiveness for stroke survivors (Hsieh et al., 2009).

Post-stroke UL motor function was assessed with the WMFT (Wolf et al., 1989). Participants were scored for functional ability on a 6-point ordinal scale as they executed 15 activities that included gross and fine motor tasks. The functional ability scores (WMFT-FAS) of the paretic arm are reported in Appendix 2. We used a 6-point ordinal scale, where 0 indicated “does not attempt with the involved arm” and five indicated “arm does participate; movement appears to be normal.” The clinometric properties of the WMFT have been ascertained in stroke (Wolf et al., 2005).

The FIM consisted of 18 items grouped into six subscales measuring self-care, sphincter control, transfer, locomotion, communication, and social cognition ability (Appendix 3) (Hamilton et al., 1987). Each item was rated from 1–7 according to the required level of assistance to perform the tasks (e.g., 1, complete assistance; 7, complete independence). A higher score on any subscale represented less disability. The FIM has good interrater reliability, construct validity, and discriminant validity (Hamilton et al., 1994).

### RS-fMRI Data Acquisition

MRIs were acquired using a 3.0-Tesla TIM Trio MRI scanner (Siemens, Erlangen, Germany). Tight but comfortable foam padding was used to minimize head movement, and earplugs were used to reduce scanner noise. RS-fMRI data were obtained using a single-shot gradient-echo sequence, echo planar imaging sequence (repetition time, 2000 ms; echo time, 30 ms; field of view, 220 mm; flip angle, 90°; matrix, 64 × 64; slice thickness, 4 mm; 36 slices/slab covering the entire brain, 180 volumes; and acquisition time, 6 min and 10 s). RS-fMRI data were acquired twice during a 4-week period. Participants were instructed to keep their eyes closed, to remain awake, to remain motionless, and not to think of anything in particular during the RS-fMRI scan.

### Pre-processing for RS-fMRI Data

Before the data pre-processing, we flipped the imaging data from left to right along the midsagittal line for the three participants who had lesions on the left hemisphere. For all participants, the right side corresponded to the ipsilesional hemisphere. The RS-fMRI data were preprocessed using Statistical Parametric Mapping 8 software^1^. The beginning 10 volumes from each participant were discarded to allow the signal to reach T1 equilibrium and participants to adapt to the scanning noise. The remaining 170 volumes were first processed for slice timing and realigned to the middle volume to correct for interscan head motions. No participants had a maximum displacement of >2 mm or a maximum rotation of >2.0°. The remaining data set was spatially normalized to the Montreal Neurological Institute echo planar imaging template and resampled into 3 × 3 × 3 mm^3^ voxels. Thereafter, nuisance variables, including the averaged signals of the ventricular, white matter, and the whole brain, and Friston 24 regressors (Friston et al., 1996) were regressed out from the fMRI data.

Next, a band-pass frequency filter (0.01–0.08 Hz) was applied to reduce low-frequency drift and high-frequency noise. Finally, the filtered blood oxygen level—dependent images were spatially smoothed using an isotropic Gaussian kernel of 8 mm full width at half maximum.

### RS-FC Analysis

RS-FC analyses were conducted using the Data Processing Assistant for Resting-State fMRI toolbox (Yan and Zang, 2010). We defined two regions of interest (ROIs; ipsilesional and contralesional M1) individually for each participant using a combination of anatomical and functional criteria (Fan et al., 2014). The anatomical M1 was defined to include voxels covering approximately the caudal half of the precentral gyrus along the anterior wall of the central sulcus, based on the Harvard–Oxford Atlas in the standard space. We overlaid each participant’s statistical parameter map for post-treatment vs. pre-treatment fMRI scans on his or her high-resolution anatomical scan and chose all active voxels within a radius of 10 mm around particular anatomical landmark.

The defined seed masks were used as the ROIs to perform the ROI-based RS-FC analysis. For each participant, the Pearson correlation coefficient between the mean time series of the ipsilesional and contralesional M1 was computed. A Fisher *r*-to-*Z* transform was used on the correlation coefficient value of each participant to improve the normality of the correlation coefficient.

In addition to the ROI approach, a whole-brain voxel-by-voxel analysis was performed for each participant using the average time course from the ipsilesional M1 as the signal of interest in a general linear model. A within-group voxel-based analysis across participants was then conducted using a general linear mixed model after transformation of the image data into the standardized space of the Montreal Neurological Institute. This analysis generated whole-brain connectivity maps for pre-treatment and post-treatment as well as a difference map between pre-treatment and post-treatment. The individual’s sex and age were entered as covariates of no interest. All maps were generated at a family-wise error-corrected *p* value of 0.05 and an extent threshold of *κ* > 10 voxels.

### Statistical Analysis

One sample *t*-tests against zero were performed on the whole-brain connectivity maps to detect the brain regions showing significant functional connectivity with the ipsilesional M1 before and after treatment. Paired *t*-tests were then conducted to identify changes on whole-brain connectivity maps between pre-treatment and post-treatment. A paired Wilcoxon test was used to identify differences between pre-treatment and post-treatment scores on the FMA-UL, WMFT-FAS, FIM, and M1-M1 connectivity values. Differences (%) between pre-treatment and post-treatment scores [(post-treatment—pre-treatment)/(post-treatment + pre-treatment)] on the FMA-UL, WMFT-FAS, FIM, and M1-M1 functional connectivity were used to estimate the changes in motor and functional outcomes as well as interhemispheric RS-FC from pre-treatment to post-treatment. The Spearman correlation test was performed to examine relationships between the RS-FC and motor and functional recovery.

To assess the relationship between the changes of interhemispheric RS-FC and motor performance, motor function, and ADL recovery after RBAT, correlation analyses were performed to identify whether the pre-to-post difference in M1-M1 connectivity was significantly associated with changes in the FMA-UL score, WMFT-FAS score, and FIM total score.

## Results

### Clinical Measures

The results of the FMA-UL, WMFT-FAS, and FIM are presented in Table 1. All participants had substantial deficits in motor performance, functional use of the ULs, and daily function before treatment.

The results showed that there were significant differences between pre-treatment and post-treatment at the corrected level of significance (*p* < 0.017) on all clinical measures. The paired Wilcoxon test on the FMA-UL total scores revealed that participants showed significant improvements in levels of motor impairment from pre-treatment to the end of RBAT (*Z* = 2.82, *p* = 0.005). Moreover, the WMFT-FAS and FIM data indicated that eligible participants had better motor function (*Z* = 2.81, *p* = 0.005) and functional independence (*Z* = 2.80, *p* = 0.005) after RBAT relative to pre-treatment.

### Functional Connectivity Results

The paired Wilcoxon test on the value of the M1-M1 RS-FC showed that participants had significantly increased M1-M1 functional connectivity from pre-treatment to the end of RBAT (*Z* = 2.80, *p* = 0.005).

**Figure 1:**
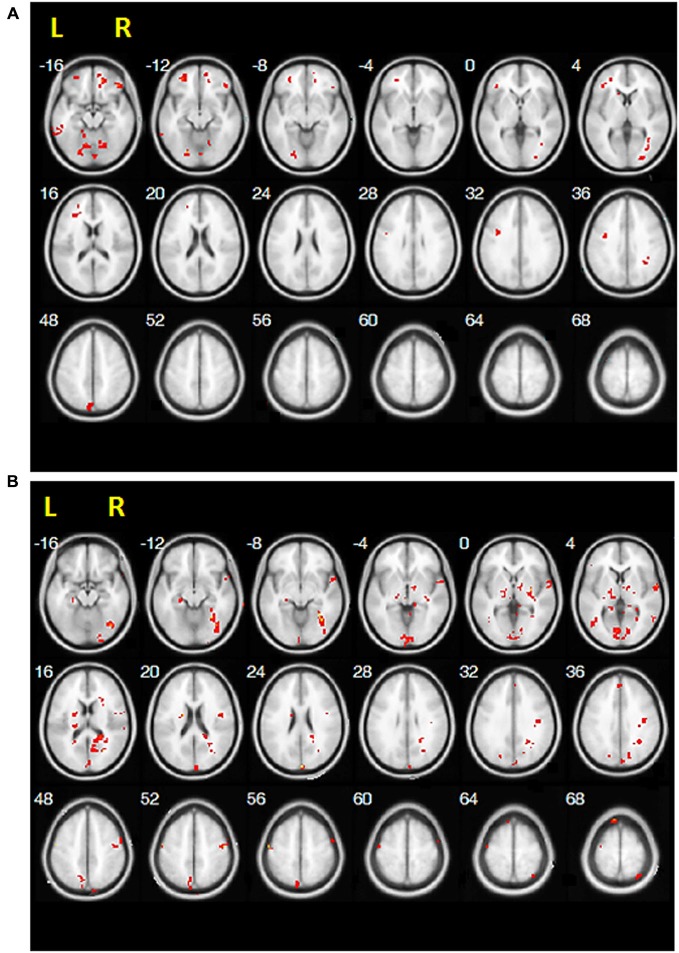
**Positive resting-state functional connectivity results of one-sample** *t*-**test for (A) pre-treatment and (B) post-treatment**.

A one sample *t*-test showed that for the ipsilesional M1 pre-treatment, participants had positive RS-FC with the bilateral middle frontal gyrus, bilateral cerebellum, bilateral inferior frontal gyrus, bilateral thalamus, ipsilesional angular gyrus, ipsilesional posterior cingulate cortex, ipsilesional superior frontal gyrus, contralesional M1, contralesional caudate nucleus, and contralesional precuneus. Moreover, negative RS-FC was observed before treatment between the ipsilesional M1 and the bilateral middle temporal gyrus, ipsilesional somatosensory cortex ipsilesional SMA, ipsilesional insula, ipsilesional superior parietal lobule, and contralesional M1 (Figure 1A and Table 2). Upon completion of RBAT, positive RS-FC with the ipsilesional M1 was seen in the bilateral somatosensory cortex (SI/SII), bilateral posterior cingulate cortex, bilateral cerebellum, bilateral thalamus, ipsilesional SMA, ipsilesional middle temporal gyrus, contralesional M1, contralesional inferior frontal gyrus, contralesional caudate nucleus, contralesional medial prefrontal cortex, contralesional anterior cingulate cortex (ACC), and contralesional middle frontal gyrus. However, participants had negative RS-FC between the ipsilesional M1 and the ipsilesional inferior frontal gyrus, ipsilesional middle frontal gyrus, ipsilesional superior frontal gyrus, contralesional temporal pole, contralesional inferior temporal gyrus, and contralesional insula after RBAT (Figure 1B and Table 2).

**Table 2:** **Regions showing significant positive and negative functional connectivity before and after treatment**.

	MNI coordinatesBrain area				
	*x*	*y*	*z*	*Z* score	Cluster size (mm^3^)
*Pre-treatment: positive correlations*
Superior parietal lobule	−21	−42	57	6.55	67
Cerebellum, uvula	21	−72	−24	5.91	14
Inferior frontal gyrus	12	36	−18	5.88	66
Cerebellum, declive	−12	−66	−18	5.84	194
Cerebellum, dentate	15	−60	−21	5.53	206
Middle frontal gyrus	−15	42	−21	5.30	12
Middle frontal gyrus	45	36	−15	5.25	26
Inferior frontal gyrus	−33	30	12	5.02	66
Angular gyrus	33	−57	39	4.83	22
Caudate nucleus	18	−3	27	4.74	11
Thalamus	15	−36	9	4.35	11
Thalamus	−6	−15	15	4.31	14
Precentral gyrus	−36	−3	42	4.29	19
Caudate nucleus	−15	21	6	4.12	68
Precuneus	−12	−51	30	4.10	13
Inferior temporal gyrus	−36	−51	−12	3.95	11
Posterior cingulate	3	−39	9	3.94	13
Superior frontal gyrus	12	57	−9	3.92	66
*Pre-treatment: negative correlations*
Precentral gyrus	48	−3	6	6.61	92
Superior parietal lobule	18	−48	57	6.54	44
Somatosensory cortex	18	−39	60	5.47	44
Primary motor cortex	−36	−15	60	5.42	63
Superior frontal gyrus	9	51	48	5.25	75
Middle temporal gyrus	63	−33	12	5.21	28
Superior temporal gyrus	−48	−6	−6	5.09	142
Middle frontal gyrus	39	−3	54	5.02	26
Hippocampus	27	−33	−6	4.65	40
Precuneus	9	−51	69	4.57	44
Insula	33	18	−12	4.44	92
Supplementary motor area	6	18	57	4.34	31
Middle temporal gyrus	−54	−75	6	4.23	45
Anterior cingulate cortex	0	48	15	4.11	43
*Post-treatment: positive correlations*
Somatosensory cortex	−54	−12	54	6.35	35
Superior temporal gyrus	63	−3	9	6.09	81
Cerebellum, declive	33	−57	−9	6.07	74
Posterior cingulate cortex	27	−66	15	6.02	526
Somatosensory cortex	51	−12	51	5.47	61
Caudate nucleus	−18	−6	21	5.44	15
Lentiform nucleus	30	−9	0	5.34	19
Parahippocampal gyrus	−12	−39	−6	5.14	32
Middle frontal gyrus	−24	9	33	5.10	11
Thalamus	−21	−27	15	4.84	13
Middle temporal gyrus	63	−66	6	4.71	14
Posterior cingulate gyrus	−24	−54	12	4.60	32
Primary motor cortex	42	−15	39	4.58	61
Middle temporal gyrus	−45	−66	6	4.45	24
Inferior frontal gyrus	−45	33	9	4.40	14
Supplementary motor area	6	27	66	4.39	11
Inferior temporal gyrus	48	−39	−18	4.37	12
Medial prefrontal cortex	−3	42	39	4.21	11
Cerebellum	−30	−36	−33	3.44	18
Thalamus	6	−6	3	3.30	21
*Post-treatment: negative correlations*
Temporal pole	−48	15	−21	5.85	32
Inferior temporal gyrus	−48	−15	−33	5.35	39
Inferior frontal gyrus	48	21	18	5.01	124
Insula	−30	15	−12	4.88	32
Middle frontal gyrus	39	57	15	4.82	91
Superior frontal gyrus	18	−9	57	4.29	37
Supramarginal gyrus	51	−45	33	4.27	33

**Figure 2:**
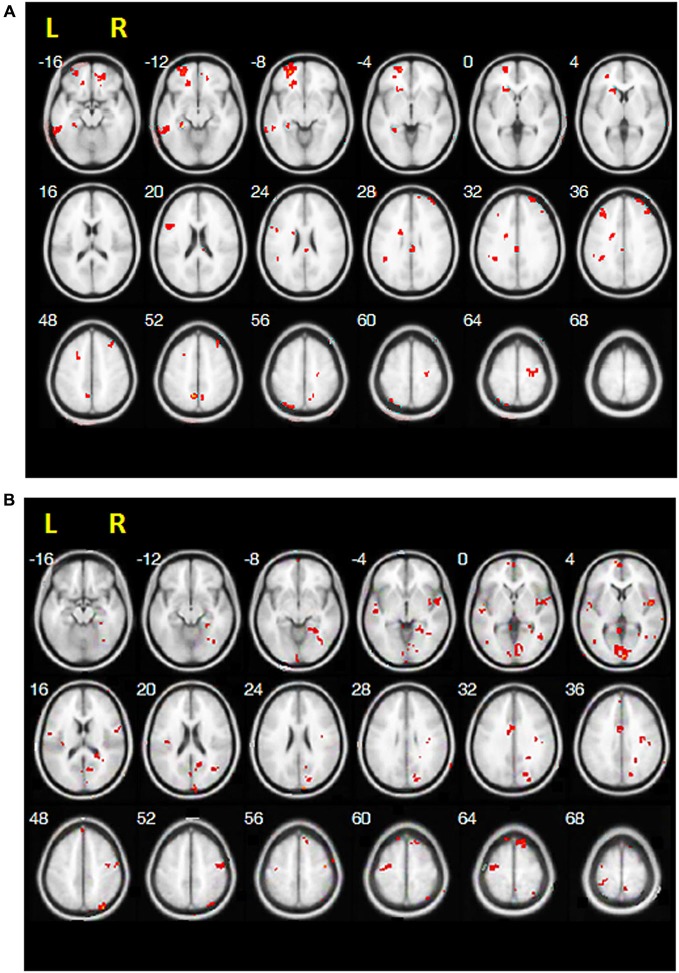
**Resting-state functional connectivity results of paired** *t***-tests between (A) pre-treatment vs. post-treatment and (B) post-treatment vs. pre-treatment**.

Figure 2 shows the maps exhibiting significant differences in RS-FC between pre-treatment and post-treatment. These brain regions are summarized in Table 3. When compared with post-treatment, greater RS-FC of the ipsilesional M1 with contralesional-lateralized brain regions was observed before treatment (Figure 2A). In contrast, increases in RS-FC were observed between the ipsilesional M1 seed and bilateral medial prefrontal cortex, bilateral M1, bilateral cerebellum, bilateral superior temporal gyrus, ipsilesional middle temporal gyrus, ipsilesional inferior parietal lobule (IPL), ipsilesional SMA, ipsilesional posterior cingulate cortex, ipsilesional SI/SII, ipsilesional caudate nucleus, contralesional ACC, contralesional insula, and contralesional middle occipital gyrus after RBAT relative to pre-treatment (Figure 2B).

**Table 3:** **Regions showing the significant differences in resting-state functional connectivity between pre-treatment and post-treatment**.

	MNI coordinates				
Brain area	*x*	*y*	*z*	*Z* score	Cluster size (mm^3^)
*Pre-treatment > Post-treatment*
Superior frontal gyrus	−21	57	−6	5.99	128
Superior temporal gyrus	−48	15	−21	5.83	13
Precuneus	−12	−57	42	5.76	29
Posterior cingulate gyrus	−2	−27	30	5.69	35
Inferior temporal gyrus	−51	−36	−18	5.68	75
Middle frontal gyrus	−30	42	6	5.54	17
Inferior parietal lobule	−39	−42	39	5.26	32
Middle temporal gyrus	−57	−54	9	5.25	11
Cerebellum, uvula	21	−75	−24	5.24	43
Superior frontal gyrus	24	57	36	5.22	92
Inferior frontal gyrus	−45	3	24	5.01	19
Caudate nucleus	−21	−3	27	4.72	27
Lentiform nucleus	−21	18	9	4.00	51
*Post-treatment > Pre-treatment*
Medial prefrontal cortex	−9	63	3	5.90	13
Superior temporal gyrus	51	−3	3	5.60	73
Inferior parietal lobule	45	−57	18	5.27	17
Primary motor cortex	−36	−15	60	5.22	30
Superior temporal gyrus	−54	−15	0	5.22	14
Supplementary motor area	18	27	63	5.07	34
Anterior cingulate gyrus	−6	6	33	5.02	26
Superior parietal lobule	36	−57	66	5.00	29
Caudate nucleus	24	−42	15	4.59	23
Posterior cingulate gyrus	24	−45	30	4.53	16
Parahippocampal gyrus	18	−48	−6	4.44	38
Somatosensory cortex	54	−12	48	4.25	39
Inferior temporal gyrus	48	−60	−18	4.25	11
Middle temporal gyrus	45	−60	0	4.07	11
Cerebellum, culmen	−6	−48	3	4.06	16
Cerebellum, declive	33	−60	−9	3.94	13
Insula	−39	−18	21	3.86	11
Middle occipital gyrus	−45	−66	3	3.75	19
Medial prefrontal cortex	6	60	6	3.70	13

### Correlation of the RS-FC with Motor and Functional Recovery

Spearman correlation analysis showed that the pre-to-post difference in M1-M1 RS-FC was significantly positively correlated with changes in the WMFT-FAS score (*R* = 0.79, *p* = 0.006) and FIM total score (*R* = 0.92, *p* < 0.001). These indicated that participants with increased M1-M1 RS-FC after the intervention had greater gains in functional use of the affected arm and daily function. However, the relations between the pre-to-post difference M1-M1 connectivity and the changes of FMA-UL score were not significant (*R* = 0.55, *p* = 0.09).

### Mediation Analysis Results

**Figure 3:**
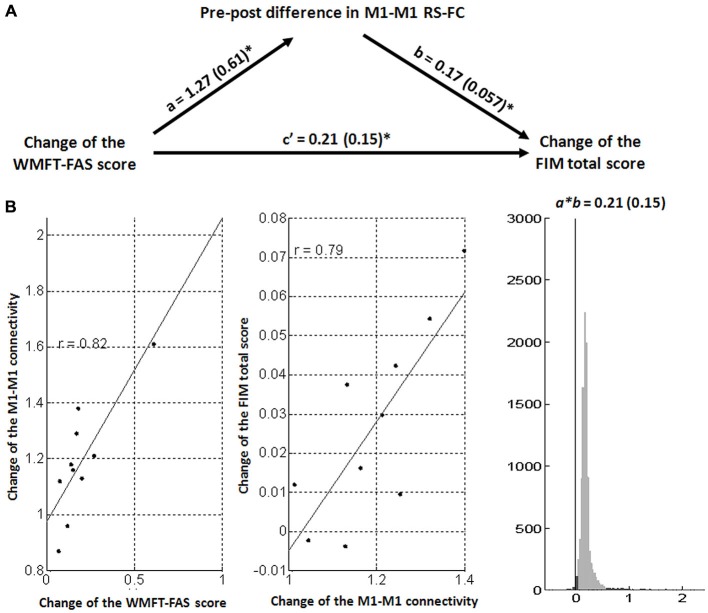
**Mediation analysis results. (A)** Path diagram shows the relationships between change scores on the WMFT-FAS and FIM from pre-treatment and the pre-to-post difference in interhemispheric M1-M1 RS-FC. The predictor region in the WMFT-FAS score is shown at the left, which predicts the M1-M1 RS-FC. This is the *a* path. The lines are labeled with path coefficients, and standard errors are shown in parentheses. The mediator factor (M1-M1 RS-FC) connection to the outcome (FIM total score) is the *b* path. This is calculated controlling for the WMFT-FAS and for the mediator factor, as is standard in mediation models. *p < 0.05, two-tailed. **(B)** Partial regression scatterplots for the (left panel) WMFT-FAS–M1-M1 RS-FC and for the (center panel) M1-M1 RS-FC–FIM total score relation. The right panel shows an example of a bootstrapped mediation effect (path *a × b*) for the M1-M1 RS-FC.

On the basis of a standard three-variable path model with a bootstrap test for the statistical significance of the product *a* × *b*, a single-level version of the mediation path model was used to get further insight of linkage between the clinical measures and RS-FC. Matlab coding implementing mediation analyses, developed by Wager et al. (2009) is freely available at^2^. In all participants, the change of interhemispheric M1-M1 functional connectivity from pre-treatment to post-treatment was a significant mediator in predicting the WMFT-FIM relation. The increased change in M1-M1 connectivity was associated with greater improvements in functional use of the affected arm and daily function after the intervention (*a* = 1.27, standard error = 0.61, *p* = 0.044; *b* = 0.17, standard error = 0.057, *p* = 0.021; *a × b* = 0.21, *Z* = 2.03, *p* = 0.042; Figure 3).

## Discussion

This is the first study to demonstrate that RBAT may facilitate motor and functional recovery in participants with post-acute stroke by modulating the functional connectivity among the motor-related areas. All participants had greater gains in motor performance, motor function, and in daily function from pre-treatment to the end of RBAT. Increased M1-M1 RS-FC was shown after RBAT relative to pre-treatment. In addition, participants receiving RBAT had increased the intrahemispheric and interhemispheric RS-FC of the ipsilesional M1 for connections to the regions implicated in sensorimotor functions, spatial processing, and attention. Further, a greater improvement in the pre-to-post difference in M1-M1 connectivity was coupled with better motor and functional gains. Mediation analysis also confirmed that such interhemispheric M1-M1 interaction was a significant mediator for the relationship between the changes of WMFT-FAS scores and FIM total scores.

In agreement with previous research, motor performance and motor function were greatly improved in all participants after RBAT (Liao et al., 2012; Wu et al., 2013; Basteris et al., 2014). A significant improvement was also found for functional outcomes after RBAT compared with pre-treatment. Gains of more than 10 points were recorded on scores of the FMA in all participants, and these differences reached minimal clinically meaningful values (Arya et al., 2011). Compared with pre-treatment, each participant exhibited significantly greater improvement on UL functional use, as defined by the WMFT-FAS after treatment. These results indicated that greater motor improvements of RBAT may induce efficient UL functions. The result on ADL performance did not corroborate the findings of some previous studies (Lo et al., 2010), possibly because of the content of the robotic protocols (Basteris et al., 2014).

Our findings coincide with previous connectivity research on stroke recovery that M1-M1 RS-FC follows an evolution in the pattern of initial decrease and gradual restoration of the level of nearly normal (Carter et al., 2010; Wang et al., 2010; Park et al., 2011; Golestani et al., 2013). For ipsilesional M1 pre-treatment, participants had greater functional connectivity with the contralesional nonprimary sensorimotor regions compared with post-treatment. These results indicate that stroke lesions may induce changes in the contralesional nonprimary sensorimotor networks for supporting the imbalance of RS-FC between motor cortices during the early stage of stroke (Puh et al., 2007). After RBAT, participants had a greater interhemispheric M1-M1 connectivity compared with pre-treatment. This enhanced connectivity pattern may reflect the importance of proper equilibrium across hemispheres for optimal function (Bloom and Hynd, 2005; Manson et al., 2008).

Recent evidence also reveals that repetitive bimanual movements can increase the excitability of the ipsilesional M1 to inhibit the contralesional M1, which promotes a rebalancing of mutual transcallosal connections and enhances motor and functional recovery (Simkins et al., 2013; Stinear et al., 2014). Here, a greater improvement in the pre-to-post difference in M1-M1 RS-FC was coupled with better motor and functional gains after RBAT was completed. These results lend support to the notion that bilateral arm training can remodel functional connections between sensorimotor cortices and remediate imbalances between the two hemispheres for stroke recovery and that RS-FC can demonstrate this plasticity.

This study extended previous treatment-induced RS-FC studies on unilateral robotic therapy (Sergi et al., 2011; Saleh et al., 2012; Varkuti et al., 2013) and showed changes in functional connectivity between M1 and other motor-related areas after stroke and robotic training. First, the disturbed intracortical and intercortical functional connectivity of the ipsilesional M1 was noted, as in previous RS-fMRI studies (Wang et al., 2010; Park et al., 2011; Rehme et al., 2012; Golestani et al., 2013).

Second, we found increased functional connectivity between the SMA, middle temporal gyrus, superior temporal gyrus, medial prefrontal cortex, caudate nucleus, and cerebellum with the ipsilesional M1 from pre-treatment to the end of RBAT. The connectivity patterns within these networks may reflect the adaptive processes that occur during the reorganization of motor control and increased recruitment of regions disconnected by stroke (Bosnell et al., 2011). Moreover, increased pre-to-post differences in RS-FC in the ipsilesional M1-ACC and ipsilesional M1-IPL were also demonstrated after RBAT relative to pre-treatment. The IPL is important for spatial processing, motor intention, and visual-motor integration (Desmurget et al., 2009), and the ACC is engaged in attentional processes related to self-monitored movement in right hemispheric stroke(Hanlon et al., 2005). A strengthened association of these two regions has beneficial effects on motor and functional performances because it involves brain networks for processing of motor control, movement initiation, attention, and spatial perception (Varkuti et al., 2013).

We observed the increased pre-to-post differences in M1-M1 connectivity in response to the RBAT correlated positively with gains in the WMFT-FAS score and FIM total score. These findings support those from studies that showed similar relationships between the interhemispheric RS-FC with motor and functional outcomes from stroke (James et al., 2009; Carter et al., 2010; Hsieh et al., 2014; Urbin et al., 2014; Young et al., 2014). The results of our mediation analysis further indicated that a greater improvement in the pre-to-post difference in M1-M1 functional connectivity mediates the changes of functional use of the affected arm and daily function. Motor and functional recovery may be enhanced with reactivation and reorganization of the sensorimotor networks along with therapy (Calautti and Baron, 2003). Structural and functional imaging studies also suggest that disruption of the intraregional and interregional motor cortices are related to motor impairment and limited functional recovery (Granziera et al., 2012; Dijkhuizen et al., 2014). Importantly, the degree of functional recovery after stroke was associated with reinstatement of interhemispheric neuronal signal synchronization and normalization of cortical network organization (van Meer et al., 2012). The current study, combining correlation and mediation analyses, illuminates how treatment-induced changes in RS-FC in the primary motor cortices influence motor and functional outcomes after stroke. Specifically, RBAT may produce a restoration of the interregional neural connections for modulating later recovery of stroke.

This study has some limitations that warrant consideration. First, the sample size is rather small, and the eligible participants had heterogeneous patient-related (e.g., age, sex) and lesion-related (e.g., lesion size, stroke subtype, and lesion location) characteristics. Such interindividual variation might have differential effects on behavioral and neurological outcomes. Second, the study lacked a control group such as post-stroke patients receiving other interventions. Third, the study only examined the effectiveness at the end of treatment and did not explore the effects after some delay or longer-term treatment effects. Fourth, no task-evoked fMRI was performed as a means to determine the relationship between brain activity during motor tasks and RS-FC. Furthermore, studies involving acute or post-acute stroke patients should be implemented, where spontaneous recovery will need to be considered as a potential confounding factor. A specific study design, such as delayed treatment, should be applied to clarify possible treatment-induced effects. Future studies need to include a larger number of stroke survivors, have a small degree of interindividual variation, recruit control groups for comparison, and focus on the maintenance of therapy results over time.

## Conclusion

We first examined RS-FC after *bilateral* robotic training, not only in M1-M1 but also in M1 with other motor-related areas. We also first used mediation analysis to capture the relations among intrinsic brain connections, motor performance, and functional independence after stroke rehabilitation. Increases in interhemispheric RS-FC between motor cortices were associated with better motor gains and improvements in ADLs performance after RBAT. These findings suggest that functional connectivity maps of the motor network may provide a prognostic value for motor and functional recovery after stroke and for monitoring the efficacy of rehabilitative therapies.

## Author Contributions

FY-T, conducted the experiments, analyzed the data, interpreted the results and statistical analysis, and drafted the manuscript. WC-Y, developed the experimental setup, contributed to the study conception, interpreted the results, and edited the manuscript. LH-L, contributed to the fMRI experiment and assisted in interpreting the results. LK-C, contributed to the study conception and study design and interpreted the results. WY-Y, conducted the fMRI experiments and assisted in interpreting the results. CY-L, contributed to monitoring the participants and conducted the fMRI experiments. All authors have read and approved of the manuscript.

## Conflict of Interest Statement

The authors declare that the research was conducted in the absence of any commercial or financial relationships that could be construed as a potential conflict of interest.

## Footnotes

^1^http://www.fil.ion.ucl.ac.uk/spm

^2^http://www.columbia.edu/cu/psychology/tor/

## Supplementary Material

The Supplementary Material for this article can be found onlineat: http://journal.frontiersin.org/article/10.3389/fnhum.2015.00546/abstract
