# Supplementary material for: Neuroplastic changes in resting-state functional connectivity after stroke rehabilitation
Source: Front Hum Neurosci. 2015 Oct 5;9:546. doi: 10.3389/fnhum.2015.00546 (PMC4617387; doi:10.3389/fnhum.2015.00546)
Supplement: Supplementary file 1 [file DataSheet_1.docx]

**Appendix 1: The Fugl-Meyer Upper Limb Assessment**

Item 1: Biceps reflex elicited

Item 2: Triceps reflex elicited

Item 3: Scapular elevation

Item 4: Scapular retraction

Item 5: Shoulder abduction

Item 6: Shoulder external rotation

Item 7: Elbow flexion

Item 8: Forearm supination

Item 9: Shoulder adduction with internal rotation

Item 10: Elbow extension

Item 11: Forearm pronation

Item 12: Hand to lumbar spine

Item 13: Shoulder flexion to 90° degrees with elbow extended

Item 14: Pronation–supination of forearm with elbow at 90°

Item 15: Shoulder abduction to 90° with elbow extended

Item 16: Shoulder flexion to 90°-180° with elbow extended

Item 17: Pronation–supination of forearm with elbow extended

Item 18: Normal reflex activity

Item 19: Wrist stable with elbow at 90°

Item 20: Wrist flexion-extension with elbow at 90°

Item 21: Wrist stable with elbow extended and shoulder at 30°

Item 22: Wrist flexion-extension with elbow extended and shoulder at 30°

Item 23: Wrist circumduction Hand

Item 24: Finger mass flexion

Item 25: Finger mass extension

Item 26: Hook grasp (MPs extended, PIPs and DIPs flexed)

Item 27: Lateral prehension (thumb adduction to hold paper)

Item 28: Palmar pinch (thumb to index finger to hold pencil)

Item 29: Cylindrical grasp (hold small can)

Item 30: Spherical grasp (hold tennis ball at fingertips) Coordination/Speed

Item 31: Movement without tremor

Item 32: Movement without dysmetria

Item 33: Movement with normal speed

**Appendix 2: The Wolf Motor Function Test**

Item 1: Forearm to table

Item 2: Forearm to box
Item 3: Extend elbow

Item 4: Extend elbow with weight

Item 5: Hand to table (front)

Item 6: Hand to box (front)

Item 7: Reach & retrieve

Item 8: Lift can

Item 9: Lift pencil

Item 10: Lift paper clip

Item 11: Stack checkers

Item 12: Flip cards

Item 13: Turn key in lock

Item 14: Fold towel

Item 15: Lift basket

**Appendix 3: The Functional Independence Measure**

Item 1: Eating

Item 2: Grooming

Item 3: Bathing

Item 4: Upper body dressing

Item 5: Lower body dressing

Item 6: Toileting

Item 7: Bladder management

Item 8: Bowel management

Item 9: Bed to chair transfer

Item 10: Toilet transfer

Item 11: Shower transfer

Item 12: Locomotion (ambulatory or wheelchair level)

Item 13: Stairs

Item 14: Cognitive comprehension

Item 15: Expression

Item 16: Social interaction

Item 17: Problem solving

Item 18: Memory
